# First Line and Second Line Chemotherapy in Advanced Cholangiocarcinoma and Impact of Dose Reduction of Chemotherapy: A Retrospective Analysis

**DOI:** 10.3389/fonc.2021.717397

**Published:** 2021-11-10

**Authors:** Christian Möhring, Jan Feder, Raphael U. Mohr, Farsaneh Sadeghlar, Alexandra Bartels, Robert Mahn, Taotao Zhou, Milka Marinova, Georg Feldmann, Peter Brossart, Martin von Websky, Hanno Matthaei, Steffen Manekeller, Tim Glowka, Jörg C. Kalff, Tobias J. Weismüller, Christian P. Strassburg, Maria A. Gonzalez-Carmona

**Affiliations:** ^1^ Department of Internal Medicine I, University Hospital, Bonn, Germany; ^2^ Department of Radiology, University Hospital, Bonn, Germany; ^3^ Department of Internal Medicine III, University Hospital, Bonn, Germany; ^4^ Department of Visceral Surgery, University Hospital, Bonn, Germany

**Keywords:** bile duct carcinoma, cholangiocellular carcinoma, first line palliative chemotherapy, second line palliative chemotherapy, retrospective analysis, gemcitabine/cisplatin, FOLFIRI

## Abstract

**Objective:**

Prognosis of patients with irresectable cholangiocarcinoma is still poor. The ABC-02 trial established the current first line (1L) standard systemic chemotherapy (CT) with gemcitabine/platinum derivate for advanced cholangiocarcinoma. However, the majority of patients needed therapy adaptions. Thus, the aim of this study was to evaluate 1L and second line (2L) therapy regimens and the impact of therapy adaptions in an unselected real-life cohort of patients with advanced cholangiocarcinoma.

**Materials and Methods:**

This is a single institution retrospective analysis of patients with irresectable cholangiocarcinoma who were treated with gemcitabine/platinum derivate from 2010 to 2018. Overall survival (OS), progression-free survival (PFS) and toxicity were analyzed for all patients, especially with regard to CT de-escalation.

**Results:**

Fifty-eight patients receiving gemcitabine/platinum derivate were included in the analysis. Median OS and PFS were 12.2 and 6.9 months. Interestingly, 41 patients (71%) needed therapy de-escalation. However, despite reduced CT exposition, there was no-significant difference in OS (10.8 months vs. 15.6 months, p = 0.127), and patients suffered from less adverse events during CT. 21 (36%) patients reached 2L CT, most often with FOLFIRI (57%). Survival beyond the end of 1L CT was 7.1 months with 2L CT vs. 2.9 months with BSC.

**Conclusion:**

In our study, the combination of gemcitabine/platinum derivate showed similar OS and PFS as randomized prospective phase II/III trials. Therapy regimen adaptions were needed in the majority of patients. However, individualized modifications of the therapy regimen allowed better tolerance as well as continuation of therapy and did not significantly influence median OS. Furthermore, our study revealed a potential survival benefit with 2L CT for selected patients.

## Introduction

Biliary tract cancer (BTC), including intrahepatic and extrahepatic cholangiocarcinoma (iCCA, eCCA) and gallbladder carcinoma, represents about 3% of all gastrointestinal malignancies. In the Western world, BTC is a rare disease with an incidence of 2-3/100,000 cases per year, but mortality rates have been increasing in the last decades, in line with increasing incidence of iCCA (1–4). The only curative treatment is radical surgery (5). However, at diagnosis, most patients already present an advanced or metastatic stage and tumor resection is debarred. Moreover, high rates of disease recurrence contribute to a poor overall prognosis (6).

In recent years, very promising information on the molecular classification and discovery of actionable mutations and genetic alterations in cholangiocarcinoma was published, enabling novel personalized therapeutic options for some patients. Therefore, the phase III study, CLARIDHY, and the phase II study, FIGHT-202, show promising results in patients with iCCA with IDH-1 mutation or FGFR-2-fusions or rearrangements, respectively (7, 8). However, the majority of patients with advanced cholangiocarcinoma is negative for these biomarkers and can only be treated with chemotherapy.

For patients with advanced cholangiocarcinoma, the phase III ABC-02 trial established the current first line (1L) systemic chemotherapy (CT) standard with gemcitabine and cisplatin. Data showed a significant benefit in overall survival (OS) for patients treated with gemcitabine and cisplatin vs. those treated with gemcitabine alone (11.7 months vs. 8.1 months, p < 0.001) (9). When contraindicated, oxaliplatin can be applied instead of cisplatin (10, 11). Evaluation of gemcitabine/cisplatin in a daily practice context is necessary to further prove the efficacy of this therapy in a non-selected group of patients with clinical characteristics different from those in the trial population. Data published to date are limited and show different outcomes (12).

Despite the presentation of the phase III trial with FOLFOX during second line (2L) therapy, to date, limited experience with 2L therapy for advanced cholangiocarcinoma after gemcitabine/cisplatin has been documented in a real world setting (13). Contrary to this phase III 2L trial, in many clinical practices, suitable patients usually receive 2L CT only when tumor progression under 1L therapy was documented or 1L CT was not tolerated. In this setting, no prospective phase III data about the effect of 2L CT has been provided to date. Only some retrospective data support the use of 2L CT as it might provide disease control for selected patients, but no regimen could prove superiority, and prognosis remains poor (14–17).

Thus, the aim of this study was to evaluate 1L and 2L therapy regimens in a real-life cohort of patients with advanced cholangiocarcinoma. Furthermore, the impact of individualized dose reduction of 1L CT on survival was analyzed in our cohort of patients.

## Materials and Methods

### Patient Characteristics

All patients diagnosed with unresectable BTC between 2010 and 2018 at the University Hospital of Bonn, Germany, were evaluated for inclusion into this study (**Table 1**). Diagnosis was based on histological (98.3%) or cytological (1.7%) validation. Therapy decision was performed in weekly multidisciplinary conferences (tumor boards) attended by representatives from the departments of oncological gastroenterology. Patients were considered inoperable because of advanced stage of disease (vascular invasion corresponding T4 stage of TNM classification or distant metastasis corresponding N2 and/or M1 stages of TNM classification), but also because of poor performance status due to severe comorbidities. Systemic CT was applied if performance status, hepatic, and renal function were considered sufficient and in accordance with the wishes of the patients.

**Table 1:** Baseline and therapy characteristics.

Parameter, Units Reference interval	Total (n = 58)		Non-modified gem/platinum derivate (n = 17)		Modified gem/platinum derivate (n = 41)		P-value
	N	%	N	%	N	%	
**Age**	59.5	50.75; 70	57	48; 69.5	61	51.5; 70.5	0.329
**Gender**							0.106
male	35	60.3	13	76.5	22	53.7	
female	23	39.7	4	23.5	19	46.3	
**ECOG**							0.781
0	33	56.9	9	52.9	24	58.5	
1	23	39.7	7	41.2	16	39	
2	2	3.4	1	5.9	1	2.4	
**Localization of tumor**							0.409
Bismuth 1-2	3	5.2	-	-	3	7.3	
Bismuth 3-4	24	41.4	10	58.8	14	34.1	
distal	1	1.7	-	-	1	2.4	
intrahepatic	25	43.1	6	35.3	19	46.3	
gallbladder	5	8.6	1	5.9	4	9.8	
**Staging**							0.533
locally advanced	17	29.3	4	23.5	13	31.7	
metastasis	41	70.7	13	76.5	28	68.3	
**Localization of metastasis**							0.897
no metastasis	17	29.3	4	23.5	13	31.7	
hepatic	17	29.3	6	35.3	11	26.8	
extrahepatic	21	36.2	6	35.3	15	36.6	
hepatic & extrahepatic	3	5.2	1	5.9	2	4.9	
**Malignancy grade**							**0.007**
1	8	13.8	6	35.3	2	4.9	
2	26	44.8	6	35.3	20	48.8	
3	18	31	3	17.6	15	36.6	
**Previous surgery**							0.958
none	48	82.8	14	82.4	34	82.9	
curative intended surgery	10	17.2	3	17.6	7	17.1	
**Concomitant surgery**							**0.006**
none	42	72.4	16	94.1	26	63.4	
palliative OP	15	25.9	–	–	15	36.6	
curative OP	1	1.7	1	5.9	–	–	
**Local concomitant therapies**							0.912
none	25	43.1	7	41.2	18	43.9	
RFA and/or PDT	22	37.9	7	41.2	15	36.6	
SIRT	9	15.5	3	17.6	6	14.6	
other (e.g. HIFU)	1	1.7	-	-	1	2.4	
RFA and/or PDT + SIRT	1	1.7	-	-	1	2.4	
**1L protocol**							0.166
Gem/Cis	50	86.2	13	76.5	37	90.2	
Gem/Ox	8	13.8	4	23.5	4	9.8	
**2L protocol**							0.765
FOLFIRI	12	57.1	4	23.5	8	19.5	
gemcitabine/cetuximab	4	19.0	2	11.7	2	4.9	
capecitabine/FOLFOX/ other	5	23.8	1	5.8	4	9.7	
**Medical conditions**							
aspirin intake	9	15.52	2	11.76	7	17.07	0.472
nicotine abuse	24	41.38	13	76.47	11	26.83	**0.006**
alcohol abuse	8	13.79	5	29.41	3	7.32	**0.040**
Diabetes mellitus II	12	20.69	2	11.76	10	24.4	0.240
Adiposity	14	24.14	4	23.53	10	24.4	0.613
Cholelithiasis	14	24.14	2	11.76	12	29.27	0.138
Hepatopathy	17	29.31	5	29.41	12	29.27	0.613
Nephropathy	5	8.62	1	5.88	4	9.76	0.539
Previous malignancy	10	17.24	2	11.76	8	19.51	0.384
ischemic heart disease	8	13.79	2	11.76	6	14.63	0.568
Apoplex	5	8.62	3	17.65	2	4.88	0.144
primary sclerosing cholangitis	6	10.34	–	–	6	14.63	0.111
inflammatory Bowel Disease	7	12.07	-	-	7	17.07	0.074
**Biochemical conditions**							
Neutrophil to lymphocyte ratio							0.151
<5	41	70.7	10	58.8	31	75.6	
>5	16	27.6	7	41.2	9	22	
dNLR							0.429
<3	41	70.7	11	64.7	30	73.2	
>3	16	27.6	6	35.3	10	24.4	
CA19-9, U/ml 34	228	61.25; 2621.35	743.1	210.9; 3806.8	163.5	54.7; 961.33	0.642
Alkaline 40 – 130 (m) phosphatase, U/l 35-105 (f)	298.5	172.25; 489.5	362	221.5; 481	217	146; 498.5	0.982
Total bilirubin, mg/dl 1.4 (m) 0.9 (f)	0.65	0.41; 1.21	0.74	0.48; 1.26	0.61	0.35; 1.19	0.844
Albumin, mg/dl 35-52	3.56	2.94; 3.86	3.38	2.71; 3.87	3.62	2.94; 3.86	0.474
Creatinine, mg/dl 0.7-1.2 (m) 0.5-0.9 (f)	0.75	0.61; 0.91	0.78	0.62; 0.94	0.74	0.59; 0.91	0.804
INR 0.9-1.1	1	1; 1.1	1	0.9; 1	1	1; 1.1	0.107
CRP, mg/l 0-3	12.8	4.8; 37.2	18.7	3.85; 47.3	11.45	5.15; 33.13	0.959
yGT, U/l 60 (m) 40 (f)	381.5	153.25; 824	508	331.5; 1207.5	313	118.5; 789.5	0.291
AST, U/l 50 (m) 35 (f)	41.0	28; 70.75	47	33; 78	40	26; 71	0.183
ALT, U/l 50 (m) 35 (f)	39.5	24; 100	64	33; 115.5	38	21; 82.5	0.496

Numerical data are presented as median with lower and upper quartile in parentheses. Categorical data are presented as absolute frequency with relative frequency in parentheses.

ALT, alanine aminotransferase; AST, aspartate aminotransferase; CA19-9, carbohydrate antigen 19-9; CEA, carcinoembryonic antigen; CRP, C-reactive protein; dNLR, derived neutrophil to lymphocyte ratio; ECOG, Eastern cooperative oncology group performance status; FOLFIRI, folinic acid, fluorouracil, and irinotecan; γGT, gamma‐glutamyl transferase; Gem/Cis, gemcitabine and cisplatin; Gem/Ox, gemcitabine and oxaliplatin; HIFU, high intensity focused ultrasound; INR, international normalized ratio; LDH, lactate dehydrogenase; N, number; PDT, photodynamic therapy; RFA, radiofrequency ablation; SC, systemic chemotherapy; SIRT, radioembolization.

Adiposity includes every documented Body-Mass-Index ≥30; hepatopathy includes documented chronic liver disease in medical history (cirrhosis Child Pugh A‐B, steatohepatitis or chronic hepatitis B infection); nephropathy includes documented chronic kidney disease (stage II‐IV due to different etiologies in patient history). Statistically significant values are marked in bold.

### Treatment Decision

All patients included in this study received combined 1L CT with gemcitabine and cisplatin as described in the ABC-02 trial (cisplatin 25 mg/m^2^ followed by gemcitabine 1000 mg/m^2^ on days 1 and 8 every three weeks) until toxicity or progression of disease. Oxaliplatin was used instead cisplatin in patients with low renal function or worsening renal function during cisplatin therapy.

At the discretion of the attending physician, in order to reduce or to avoid increase of toxicity, de-escalation of gemcitabine/cisplatin was adapted to application of gemcitabine/cisplatin every two weeks. Especially in older and comorbid patients, decision of therapy adaption was based on CTCAE grade 1 and 2 adverse events and according to patient wishes. After progression of disease or occurrence of toxicity, 2L therapy was offered if performance status was considered sufficient and in accordance with patient wishes. The most frequently applied 2L CT was FOLFIRI (folinic acid, fluorouracil and irinotecan) (57%). Other therapy regimens were FOLFOX (folinic acid, fluorouracil and oxaliplatin), capecitabine (24%) or gemcitabine/cetuximab (19%).

### Data Collection and Study Design

This is a single institution retrospective analysis of patients with irresectable cholangiocarcinoma who were treated with gemcitabine/platinum derivate in 1L therapy from 2010 to 2018. Baseline parameters (**Table 1**) were recorded prior to therapy. Patients were followed until death or March 2018. When lost to follow-up, patients were censored at date of last visit. Tumor response was assessed by computer tomography and/or magnetic resonance imaging (MRI), which were performed regularly every 8-12 weeks. According to the radiologist’s evaluation, tumor response was classified as complete or partial remission (CR, PR), stable disease (SD) or progressive disease (PD) corresponding to the response evaluation criteria in solid tumors (RECIST, version 1.1). CT toxicity was recorded according to the common terminology criteria for adverse events (CTCAE, version 4.03). This study was reviewed and approved by the Ethics Committee of the Medical Faculty of the University of Bonn (No. 341/17).

### Statistical Analysis

Differences in continuous variables, expressed as medians and first and third quartiles were assessed using non-parametric Mann-Whitney test. Categorical variables, expressed as absolute frequencies and percentages, were compared using Pearson’s chi squared test or Fisher’s exact tests. Survival was compared by log-rank test and transcribed into Kaplan-Meier diagrams. OS, defined as the time period from application of first tumor-specific therapy until death. PFS, defined as the time period from beginning first line chemotherapy or second line chemotherapy until disease progression or death.

Uni- and multivariate analyses were performed using Cox regression models. Variables that showed significant p-values in univariate analysis were included in multivariate backward conditional Cox regression analysis. P-values ≤ 0.05 were considered statistically significant. SPSS version 22 (IBM Corporation, Armonk, NY, USA) was used for statistical analysis.

## Results

### Patient Characteristics at Base Line

**Figure 1:**
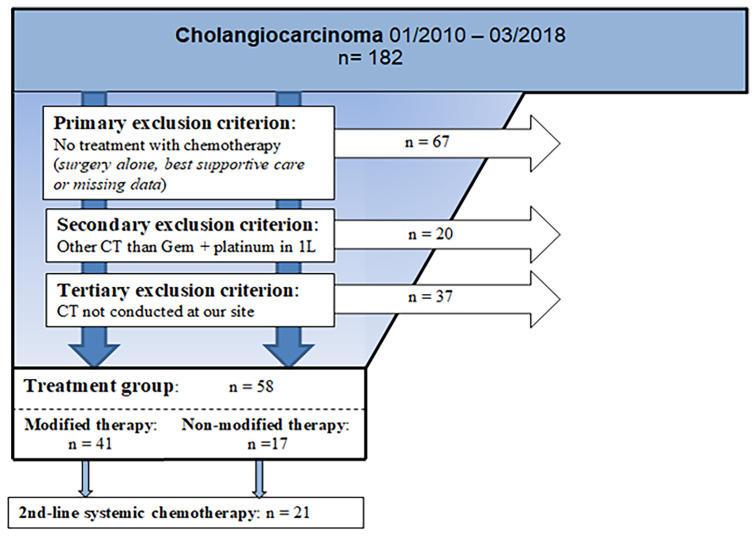
Flowchart of inclusion criteria. N, number; Gem, gemcitabine; CT, chemotherapy.

A total of 182 patients with unresectable BTC was identified. Of this total, 131 (72%) received systemic chemotherapy, and of these, 105 received a combination of gemcitabine with platinum derivate as palliative 1L therapy. Of these 105 patients, 58 fulfilled our inclusion criteria for evaluation in this study (47 patients performed the therapy outside of our hospital with missing data) (s. **Figure 1**, flow chart).

Patient characteristics of these 58 patients are shown in **Table 1**. Median age at diagnosis was 59.5 years and 35 patients (60%) were male. eCCA was diagnosed in 48%, iCCA in 43%, and 9% of patients presented gallbladder carcinoma. At the time of diagnosis, 41 patients (70.7%) presented metastasis, with 29% being hepatic metastasis, 36% extrahepatic metastasis and 5% hepatic and extrahepatic metastasis. Interestingly, ten patients (17%) had a previous non-biliary malignancy which was considered cured, implying a remission-free interval of at least five years after complete tumor remission. Six patients (10%) had a history of primary sclerosing cholangitis (PSC), a known risk factor for BTC, particularly at a young age. The vast majority of patients was fit, with 97% presenting an Eastern cooperative oncology group (ECOG) performance status of 0 or 1.

### Treatment Characteristics

Seventeen patients (29%) completed at least eight cycles of chemotherapy according to the ABC-02 protocol, while the majority of patients (41 patients, 71%) required therapy reductions with modification in timing and sometimes additional dosage reduction of CT in the first cycles of CT (s. **Table 1**). Reasons for dosage modification were especially adverse events and in particular, hematologic toxic effects. The baseline characteristics of patients requiring de-escalation were similar to those of patients, who tolerated the standard therapy. However, risk factors, such as nicotine and alcohol abuse, were more frequent in the group of patients treated with unmodified CT than in the group that required a modified/reduced CT regimen. Interestingly, patients in the non-modified CT group had tumors with lower malignancy grading. Concomitant surgical therapy was performed in 27.5% of all patients and was significantly different between the two groups of patients: only patients treated according to modified and reduced CT protocol needed palliative surgery (25.9%), while this was not required by any of the patients treated according to the unaltered ABC-02 protocol (p = 0.010). By contrast, only one patient underwent surgery with an initial curative intention, and this patient was treated according to the unaltered ABC-02 protocol. Additional to systemic CT, 33 (57%) patients received at least one locoregional treatment, including photodynamic therapy (PDT), radiofrequency ablation (RFA) or radioembolization, with equal distribution in both groups (p = 0.910). No significant differences in the additional locoregional treatments between the two groups of patients were observed.

### Efficacy of 1L Therapy With Gemcitabine/Platinum Derivate

**Figure 2:**
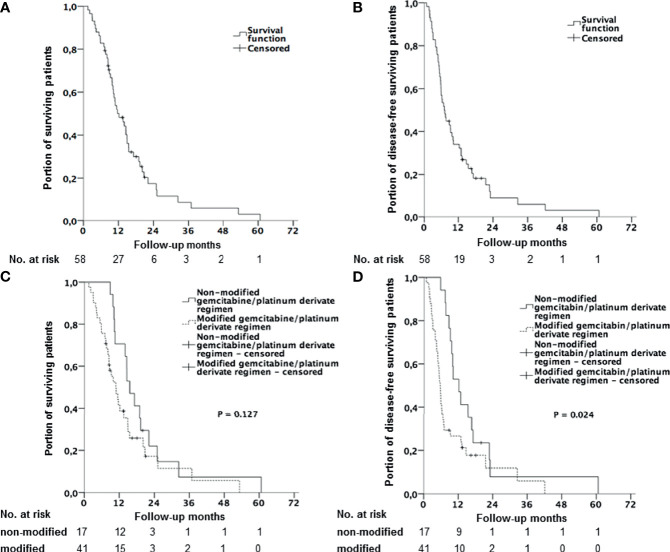
Kaplan-Meier curves with log-rank p. **(A)** Overall survival (OS) for all patients receiving combined 1L CT with gemcitabine and platinum derivate. **(B)** Progression free survival (PFS) for all patients receiving a combined 1L CT with gemcitabine and platinum derivate. **(C)** Overall survival (OS) stratified by therapy regimen (gemcitabine/platinum derivate without dose reduction vs. modified/reduced gemcitabine/platinum derivate). **(D)** Progression free survival (PFS) stratified by therapy regimen (gemcitabine/platinum derivate without dose reduction vs. modified/reduced gemcitabine/platinum derivate).

The median OS for the whole study population was 12.2 months (95% CI: 8.51, 15.89) and median progression free survival (PFS) was 6.9 months (95% CI: 5.07, 8.80) (s. **Figures 2A, B**). Ten patients (17%) reached, as best response, PR, 31 (54%) SD and 13 (22%) progressed under 1L CT. Thus, overall response rate (ORR) was 17% and disease control rate (DCR) was 71%.

While the primary tumor localization did not seem to have any statistically significant influence on OS, patients with gallbladder cancer [22.2 months (95% CI: 0.65, 43.68)] had a longer OS than patients with eCCA [14.5 months (95% CI: 8.65, 20.42)] or iCCA (10.8 months (95% CI: 8.42, 13.18) (p for log-rank = 0.190).

Interestingly, median OS for patients who required protocol reduction was slightly reduced to 10.8 months (95% CI: 7.72, 13.88) vs. 15.6 months (95% CI: 12.03, 19.11) for patients who received CT according to the unaltered ABC-02 protocol. However, this difference seems to be not significant (HR 1.61; 95% CI: 0.87, 2.98; p = 0.127) (**Figure 2C**). PFS was reduced to 5.6 months (95% CI: 4.81, 6.40) compared to 12.3 months (95% CI: 8.61, 16.05) in the group with unaltered CT protocol (HR 1.99; 95% CI: 1.08, 3.66; p = 0.024), (**Figure 2D**). Disease control was achieved more often with the unaltered CT protocol than in the modified group (94% vs. 61%, p = 0.012), while no difference in ORR was observed (24% vs. 15%, p = 0.458).

In order to clarify the role of adherence to the CT protocol in survival and to stratify further possible independent predictors for survival, univariate and multivariate analysis of baseline, tumor and therapy characteristics were performed. As shown in **Table 2**, in the univariate analysis, some parameters were identified as predictors of survival, including gGT, neutrophile-to-lymphocyte ratio, LDH, albumin and CA 19-9-level over 200 U/ml. In the multivariate analysis, LDH (HR 1.007, 95% CI 1.003, 1.010, p < 0.001) and gGT (HR 1.001, 95% CI 1.000, 1.001, p = 0.029) were identified as independent factors influencing overall survival (**Table 2**). Reduction of CT was not found to be a negative predictor of survival.

**Table 2:** Univariate and Multivariate Analysis.

Univariate	Reference interval		P-Value	HR	95% CI for HR		
					lower	upper	
Male gender			0.143	1.597	0.853	2.992	
Age			0.276	0.986	0.961	1.011	
Extrahepatic CCA			0.212		Reference		
Intrahepatic CCA			0.493	1.232	0.679	2.235	
Gallbladder carcinoma			0.159	0.414	0.121	1.414	
Metastatic disease			0.454	1.279	0.671	2.438	
Histological grading 1			0.245		Reference		
Histological grading 2			0.651	0.823	0.353	1.917	
Histological grading 3			0.382	1.470	0.620	3.489	
CA19-9 >200 IU/L			**0.039**	1.897	1.034	3.481	
Alkaline phosphatase			0.732	1.000	0.999	1.001	
LDH (U/L)	250		**< 0.001**	1.006	1.003	1.009	
Total bilirubin, mg/dl	1.4 (m) 0.4 (f)		0.738	0.979	0.862	1.111	
Serum albumin, mg/dl	35-52		**0.049**	1.180	1.001	1.391	
Creatinine, mg/dl	0.7-1.2 (m) 0.5-0.9 (f)		0.306	0.434	0.088	2.146	
CRP, mg/ml	0-3		0.064	1.007	1.000	1.015	
AST, U/l	50 (m) 35(f)		0.631	1.001	0.996	1.007	
ALT, U/l	50 (m) 35 (f)		0.922	1.000	0.996	1.004	
yGT, U/l	60 (m) 40 (f)		**0.048**	1.001	1.000	1.001	
Leucocytes, 10^3/µl	3.9-10.2		0.082	1.112	0.987	1.254	
Neutrophile to lymphocyte ratio			**0.001**	1.121	1.046	1.201	
ECOG 0			0.202	Reference			
ECOG 1			0.083	1.696	0.933	3.082	
ECOG 2			0.912	0.921	0.215	3.940	
Nicotine abuse			0.454	0.799	0.445	1.437	
Metal stenting			0.081	2.310	0.903	5.911	
Ethanol abuse			0.545	1.271	0.585	2.763	
PSC			0.403	1.447	0.609	3.439	
Any local therapy			0.762	0.915	0.516	1.623	
GemOx as 1L			0.220	0.574	0.237	1.394	
Modification of 1L CT			0.130	1.609	0.869	2.980	
2L CT treatment			0.942	0.979	0.545	1.758	
**Multivariate**						
LDH (U/L)	250		**<0.001**	1.007	1.003	1.010	
yGT U/l	60(m) 40(f)		**0.029**	1.001	1.000	1.001	
Serum albumin mg/dl	35-52		0.071	1.166	0.987	1.377	

1L, first line; ALT, alanine aminotransferase; AST, aspartate aminotransferase; CCA, cholangiocellular carcinoma; CRP, C-reactive protein; CT, chemotherapy; ECOG, Eastern cooperative oncology group performance status; γGT, gamma‐glutamyl transferase; Gem/Ox, gemcitabine and oxaliplatin; LDH, lactate dehydrogenase; PSC, primary sclerosing cholangitis. Statistically significant values are marked in bold.

**Figure 3:**
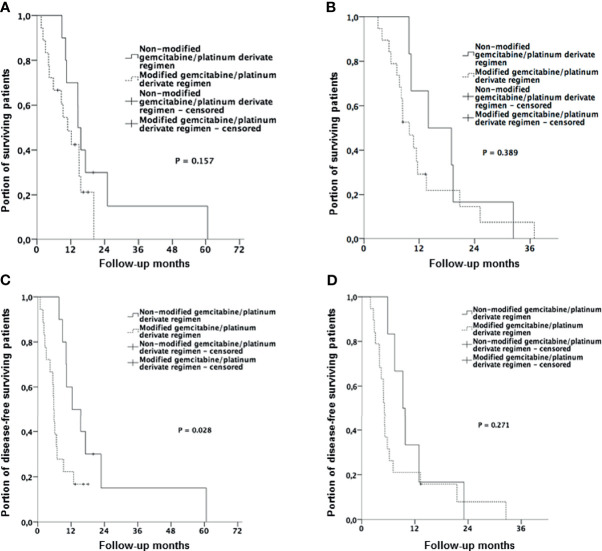
Kaplan-Meier curves with log-rank p for different tumor localizations. **(A)** Overall survival (OS) in patients with extrahepatic cholangiocarcinoma stratified by therapy regimen (gemcitabine/platinum derivate without dose reduction vs. modified/reduced gemcitabine/platinum derivate) **(B)** Overall survival (OS) in patients with intrahepatic cholangiocarcinoma stratified by therapy regimen (gemcitabine/platinum derivate without dose reduction vs. modified/reduced gemcitabine/platinum derivate). **(C)** Progression free survival (PFS) in patients with extrahepatic cholangiocarcinoma stratified by therapy regimen (gemcitabine/platinum derivate without dose reduction vs. modified/reduced gemcitabine/platinum derivate). **(D)** Progression free survival (PFS) in patients with intrahepatic cholangiocarcinoma stratified by therapy regimen (gemcitabine/platinum derivate without dose reduction vs. modified/reduced gemcitabine/platinum derivate).

**Figure 3** shows the results of a subgroup analysis of patients suffering from eCCA and iCCA. Accordingly, in patients suffering from eCCA, CT protocol modification had no statistically significant influence on median OS (14.6 months vs. 10.9 months, p = 0.157). However, a statistically significant longer median PFS (12.3 months vs. 5.7 months, p = 0.028) was found in patients where CT protocol was maintained, than in patients with modified CT. By contrast, no influence of CT regimen modification on OS and PFS was observed in patients with iCCA.

### Efficacy of 2L Therapy

**Figure 4:**
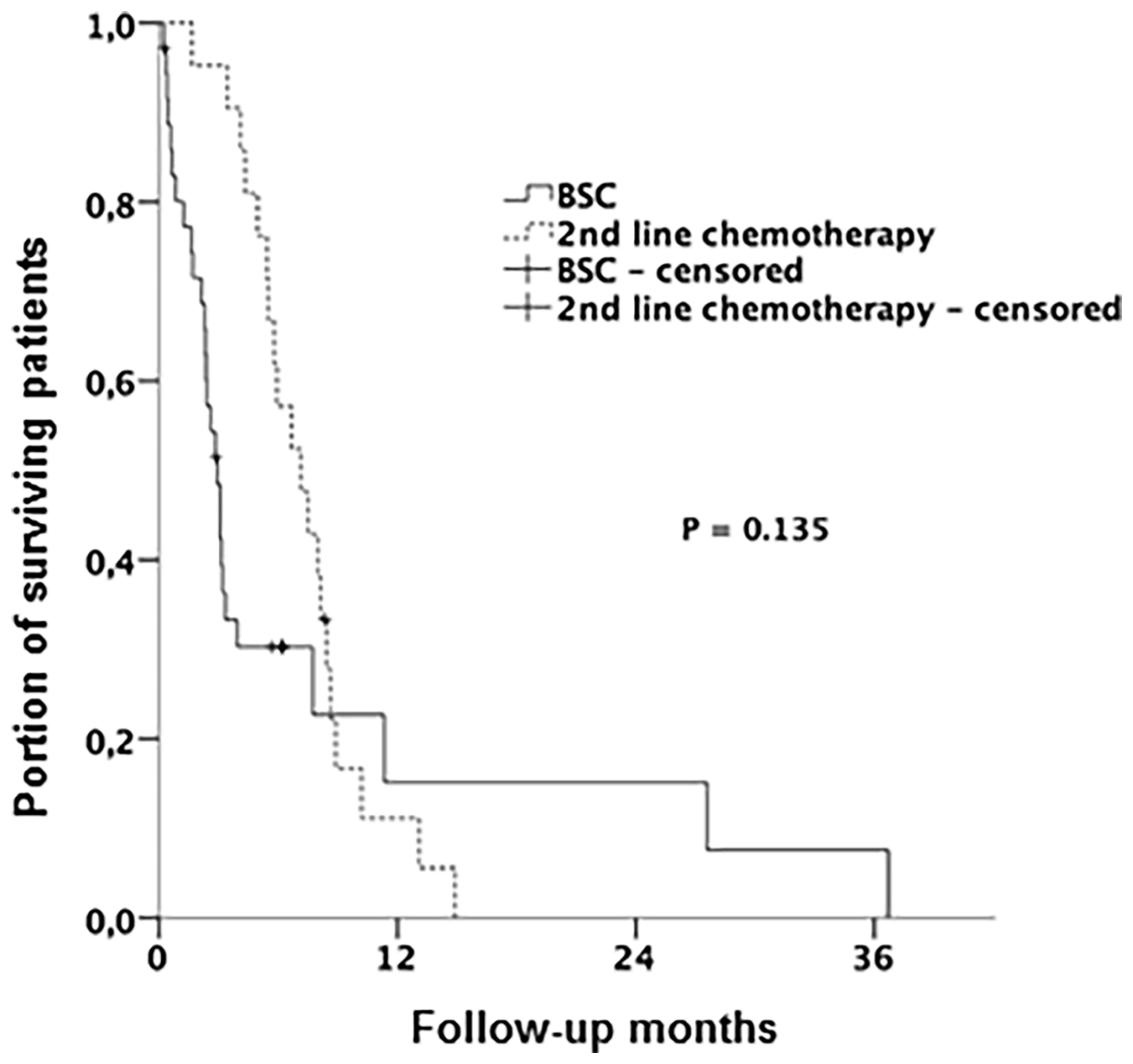
Kaplan-Meier curves with log-rank p. Overall survival (OS) stratified by therapy regimen (receiving 2nd line CT vs. BSC). BSC, best support care; CT, chemotherapy.

Twenty-one patients (36%) were fit enough to receive 2L CT. The most often applied protocol in the 2L setting was FOLFIRI (folinic acid, fluorouracil and irinotecan) (57%). Median OS (mOS) of the patients receiving 1L and 2L CT was 14.0 months, while the mOS for patients receiving only 1L was 10.2 months (HR 0.98; 95% CI: 0.55, 1.76; p = 0.458). Survival beyond the end of 1L CT was 2.9 months in patients receiving only best supportive care (BSC) vs. 7.1 months in patients receiving 2L CT (HR 0.64; 95% CI: 0.35, 1.16; p = 0.135) (**Figure 4**). There was no difference in 2L therapy-specific survival between the 2L CT therapy regimen (p for log-rank = 0.459). Best response to 2L CT was SD, which was reached in 19% (four patients). Baseline characteristics that were more frequent in the group receiving 2L CT were initial age younger than 65 years (p = 0.015) and intrahepatic or gallbladder tumor localization (p = 0.024).

### Toxicity

Distribution of adverse events (AE) is shown in **Table 3**. Hematologic toxic effects were the most frequent AEs (69%). Ten patients (17%) developed significant amounts of ascites, nine patients (16%) presented grade 3-4 thromboembolic events, while impaired renal function was seldom seen. Similar distribution of AEs was observed under 2L CT (**Table 4**). In the group with adherence to ABC-02 CT protocol, significantly more frequent grade 3 and 4 hematologic toxicities (88% vs. 61%, p = 0.041), such as decreased platelet and neutrophil counts, were documented (p = 0.042 and p = 0.026, respectively). Furthermore, significantly more frequent liver function impairment with increased alanine aminotransferase level and more biliary sepsis was detected in the group of patients treated according to the ABC-02 CT protocol (p = 0.016 and p = 0.030, respectively).

**Table 3:** Adverse events in 1L chemotherapy.

Adverse Events 1L Chemotherapy (Grade 3 - 4)		Total (n = 58)	Non-modified SC (n = 17)	Modified SC (n = 41)	P-Value
Hematologic toxic effects	Leucopenia	15 (25.9)	5 (29.4)	10 (24.4)	0.094
	Thrombopenia	24 (41.4)	11 (64.7)	13 (31.7)	**0.042**
	Anemia	27 (46.6)	9 (52.9)	18 (43.9)	0.734
	Neutropenia	23 (39.7)	11 (64.7)	12 (29.3)	**0.026**
Liver function	Increased alanine aminotransferase level	11 (19.0)	7 (41.5)	4 (9.8)	**0.016**
	Ascites	10 (17.2)	3 (17.7)	1 (5.6)	0.130
Non-hematological toxic effects	Anorexia	6 (10.3)	2 (11.8)	4 (9.8)	0.819
	Fatigue	2 (3.5)	1 (5.9)	1 (5.6)	0.513
	Nausea	6 (10.3)	2 (11.8)	4 (9.8)	0.819
	Vomiting	3 (5.2)	1 (5.9)	2 (4.9)	0.875
	Impaired renal function (GFR)	3 (5.2)	1 (5.9)	2 (4.9)	0.875
	Infection				
	without neutropenia	15 (25.9)	6 (35.3)	9 (22.0)	0.467
	with neutropenia	1 (1.7)	0	1 (5.6)	0.516
	biliary sepsis	7 (12.1)	5 (29.4)	2 (4.9)	**0.030**
	other	6 (10.3)	2 (11.8)	0	0.148
	Thromboembolic event	9 (15.5)	3 (17.7)	6 (14.3)	0.773
	Polyneuropathy	3 (5.2)	1 (5.9)	2 (4.9)	0.875
	Obstipation	1 (1.7)	0	1 (5.6)	0.516
	Diarrhea	1 (1.7)	0	1 (5.6)	0.516

Categorical data are presented as absolute frequency with relative frequency in parentheses.

N, number; GFR, glomerular filtration rate. Statistically significant values are marked in bold.

**Table 4:** Adverse events in 2L chemotherapy.

Adverse Events 2L Chemotherapy (n = 21)		Grade 1-2	Grade 3-4
Hematologic toxic effects	Leucopenia	10 (47.6)	6 (28.6)
	Thrombopenia	17 (81.0)	2 (9.5)
	Anemia	12 (57.1)	8 (38.1)
	Neutropenia	2 (9.5)	5 (23.8)
Liver function	Increased alanine aminotransferase level	18 (85.7)	0 (0)
	Ascites	3 (14.3)	6 (28.6)
Non-hematological toxic effects	Alopecia	1 (4.8)	0 (0)
	Anorexia	8 (38.1)	5 (23.8)
	Fatigue	12 (57.1)	3 (14.3)
	Nausea	3 (14.3)	1 (4.8)
	Vomiting	1 (4.8)	0 (0)
	Impaired renal function (GFR)	5 (23.8)	5 (23.8)
	Infection		
	without neutropenia	3 (14.3)	5 (23.8)
	with neutropenia	0 (0)	1 (4.8)
	biliary sepsis	0 (0)	1 (4.8)
	Thromboembolic event	3 (14.3)	1 (4.8)
	Polyneuropathy	5 (23.8)	0 (0)
	Obstipation	3 (14.3)	0 (0)
	Diarrhea	6 (28.6)	2 (9.5)

Categorical data are presented as absolute frequency with relative frequency in parentheses.

N, number; GFR, glomerular filtration rate.

## Discussion

Biliary tract cancer is a rare but increasing malignancy, which is still very difficult to treat. Surgical resection is the only curative option and at the time of diagnosis, this is suitable only for a minority of patients. For the majority of patients, systemic chemotherapy remains the current treatment of choice. Since 2010, a combination of gemcitabine and platinum derivate is the established standard 1L systemic therapy for patients with advanced cholangiocarcinoma (9–11). Data from the phase III ABC-02 showed a significant benefit in OS for patients treated with gemcitabine and cisplatin vs. those treated with gemcitabine alone (11.7 months vs. 8.1 months, p <0.001) (9). When contraindicated, oxaliplatin might be applied instead of cisplatin (10, 11). However, data evaluating the efficacy and tolerability of gemcitabine/platinum derivate in a daily practice context in non-selected patients are still sparse and showed different outcomes to date (12). Furthermore, experience with 2L therapy in patients with advanced cholangiocarcinoma after gemcitabine/cisplatin remains limited. Thus, in this retrospective study, 1L CT with gemcitabine/platinum derivate and use of 2L CT for patients with advanced cholangiocarcinoma were studied in a real-life cohort of patients. Furthermore, the impact of individualized dose reduction of 1L CT on survival was especially analyzed in our cohort of patients.

Despite this being an unselected patient cohort, gemcitabine/platinum derivate achieved similar benefit on OS with 12.2 months (95% CI: 8.51, 15.89) and on PFS with 6.9 months (95% CI: 5.07, 8.80) when compared to the data published in prospective trials (9–11). Furthermore, ORR was 17% and disease DCR was 71%. In particular, our findings are comparable to the findings of the ABC-02 trial regarding OS (11.7 months) but are slightly lower regarding PFS (6.9 vs. 8.0 months in the ABC-02 trial, respectively) or DCR (71% vs. 81.4% in the ABC-02 trial, respectively) (9). 17.1% of our patients were radically treated compared to 18.1% of the patients in the study by Valle et al. (9). This can indicate a comparable tumor burden in both groups.

One of the main reasons for the reduced PFS in comparison to the study by Valle et al. seems to be the reduced therapy exposure due to the adaption of therapy frequency to every two weeks in our cohort of patients. Moreover, 36.2% of our patients received a 2L therapy explaining a longer survival despite shorter PFS. The second prospective randomized trial, BT-22, published 2010 by Okusaka et al., showed a similar OS with 11.2 months (95% CI: 9.1-12.5), but a slightly lower outcome in PFS with 5.8 months (95% CI: 4.1-8.2) than our cohort (18).

Further phase III randomized studies have been conducted after the ABC-02 study in order to improve the efficacy of gemcitabine/cisplatin or gemcitabine/oxaliplatin as first line therapy. Unfortunately, none of these studies could show a significant benefit of additional combined therapies compared to gemcitabine/platinum derivate.

In terms of efficacy, the Japanese trial FUGA-BT including 354 patients showed slightly better mOS of 13.5 months and shorter PFS with 5.8 months than our cohort (19). The KHBO1401-MITSUBA trial also from Japan, showed similar mOS with 12.6 months and shorter PFS with 5.5 months for the patients in the gemcitabine/cisplatin arm (20). Interestingly, gemcitabine plus cisplatin showed shorter mOS with 8.3 months (95% CI=0.60-1.02) in patients with unresectable gallbladder cancer compared to the ABC-02 trial or compared to our cohort (21). Further phase III trials using gemcitabine and oxaliplatin as standard first line therapy in patients with advanced biliary tract cancer showed lower efficacy in terms of mOS and PFS than our cohort (22–24).

The use of gemcitabine/platinum derivate in unselected patients with advanced cholangiocarcinoma in clinical daily practice has been studied in several retrospective studies. A large retrospective analysis from Korea with 740 patients showed a lower (mOS of 10.4 months (95% CI:9.6-11.2), lower median PFS of 5.2 months (95% CI:4.7-5.6) and lower DCR of 60% compared to our cohort of patients (25). A multicentric retrospective study from The Netherlands with 138 patients receiving gemcitabine/cisplatin also showed lower outcomes compared to our findings: mOS of 9.6 months (95% CI:6.7-12.5) in patients meeting the ABC-02 trial criteria and mOS of 9.5 months (95% CI:7.7-11.3) in patients who did not meet the ABC-02 trial criteria.

Analysis of the baseline characteristics revealed many differences between the above-mentioned trials, which can confound comparisons of the outcomes. In our study, 48% of the patients had eCCA, 43% iCCA and only 9% of patients had gallbladder carcinoma. By comparison, in the ABC-02 trial, 35.8% of patients receiving gemcitabine/cisplatin had gallbladder carcinoma (9). In the retrospective trials mentioned above, 25% and 18.1%, respectively, of patients had gallbladder carcinoma (12, 25). From a histological, molecular and genetic point of view, gallbladder carcinoma and eCCA are different tumor entities than iCCA with different prognosis (1, 26). In accordance with the subgroup analysis in the ABC-02 trial, where patients with gallbladder carcinoma showed improved response to systemic chemotherapy with gemcitabine/cisplatin, our patients with gallbladder cancer (n = 4) had the longest mOS of all three tumor localizations with 22.2 months (9). However, all in all there was no significant difference in overall survival between the three tumor localizations in our group (p for log-rank = 0.190), supporting the findings of Kim et al. (25).

Lastly, concomitant tumor-specific local therapies may have also influenced the outcome of our patients receiving CT. For instance, there is increasing evidence that concomitant endobiliary local therapies, such as PDT and RFA, seem to have a beneficial effect on survival, especially in patients with eCCA (27). In the here presented study, 33 patients (56.9%) received concomitant local therapy with RFA, PDT or SIRT. In the ABC-02 trial, only 0.5% of the gemcitabine/cisplatin patients were concomitantly treated with PDT, while 37.3% received unspecified other therapies. A positive effect of locally applied therapies cannot be ruled out and must be studied in further trials.

In our real-life cohort of unselected patients, chemotherapy regimen was delayed to every two weeks and was sometimes additionally dose-reduced much more frequently than in the ABC-02 in order to avoid or to reduce toxicity. For instance, only 29% of our patients were able to complete the ABC-02 protocol of eight cycles of chemotherapy without doses reduction or timing delay, while in the ABC-02 trial, 55% of patients completed treatment on schedule for 24 weeks (9). This may be a main reason why PFS and DCR observed in our total cohort were lower than in the ABC-02 trial. However, while the patients in our cohort with full adherence to the ABC-02 protocol showed an even better outcome in terms of OS and PFS (15.6 and 12.3 months respectively) than patients in the ABC-02 trial, no statistically significant difference in comparison to our modified CT group was detectable for OS (10.8 months, p for log-rank = 0.127). By contrast, patients needing a modified chemotherapy protocol reached reduced PFS (5.6 months, p for log-rank = 0.024). Accordingly, in the univariate and the multivariate analysis, reduction of chemotherapy had no statistically significant influence on OS. As expected, the median age of the modified CT group was about four years above the median age of the group treated in full adherence to the ABC-02 protocol. Interestingly, nicotine or alcohol abuse in the medical history were much frequent in the patients treated according to the unmodified chemotherapy protocol and all patients with underlying PSC required modified CT regimen.

Regarding therapy exposure, our patients reached a median number of gemcitabine/platinum derivate cycles of seven (range 1-36). The study by Kim et al., with a median number of five cycles, as well as the multicentric study from The Netherlands, with a median number of six cycles, showed lower therapy exposure (12, 25). Modification of the therapy regimen may explain an increased adherence to therapy allowing longer therapy exposure, which seems to have a major benefit in terms of survival (28).

In a further subgroup analysis of patients suffering from eCCA and iCCA, patients with eCCA showed significantly shorter PFS when CT protocol was modified than patients with non-modified CT (p = 0.023). This effect was not seen in patients with iCCA and therapy modification did not affect OS in both localizations. These findings partially reflect the results of the subgroup analysis of the ABC-02 trial, which demonstrated a slightly lesser effect of gemcitabine and cisplatin in the therapy of eCCA (9). Our results additionally suggest a possible stronger dose-dependence for disease control in extrahepatic CCA localization.

Regarding toxicity assessment, we registered more adverse events, especially hematologic toxic ones, than the ABC-02 trial or the further performed phase III trials with gemcitabine plus platinum derivate as standard arm (e.g. 35.1% grade 3-4 toxicities in the FUGA-BT trial (9, 19–24). However, our data are similar to the findings reported by the prospective Japanese BT22 study, which recorded a decreased neutrophil amount in seven patients (43.8%) vs. 23 patients (39.66%) in our study. Of note, as in our patients, in the BT-22 study, chemotherapy was applied until progression of the disease, while in the ABC-02 trial, treatment was applied only until 24 weeks, explaining the lower toxicity (18). In the retrospective trial by Dierks et al., similar hematologic toxicities were observed (neutropenia in 32.8% and platelet reduction in 11.7%) (12). Non-hematologic events were comparable in all three studies. For example, fatigue, nausea or vomiting were recorded in 4.2% of patients in the BT22 study vs. 10.3% in our study vs. 4.1% in the chemotherapy in daily practice trial by Dierks et al. In our study, the most frequent reason for ending 1L CT was progression of disease (53.4%), followed by toxicity (24.1%) and early death (10.3%). Only one patient asked for termination of CT (1.7%). Interestingly, in the modified group, the number of patients, who stopped 1L CT because of adverse events, did not differ significantly to these in the group without CT modification (19.5% vs. 35.3%, p = 0.311). In the study of Kim et al., only 9% of patients discontinued chemotherapy due to treatment-related toxicities or patient’s wish. However, no information on frequency and severity of adverse events was presented (25).

Despite the presentation of the phase III ABC-06 trial with FOLFOX in 2L CT, very limited experience with 2L therapy for advanced cholangiocarcinoma after gemcitabine/cisplatin has been documented to date in a real world setting (13). In the ABC-06 study, FOLFOX as 2L CT with active symptom control (ASC) vs. ASC alone showed a significant difference in OS with 6.2 months vs. 5.3 months. However, contrary to this 2L trial from the UK, where 1L CT is currently interrupted after a maximum of six months of treatment, in our clinical practice, fit patients usually receive 1L CT until disease progression or therapy intolerance. Patients meeting the criteria will subsequently be treated with 2L CT to the end of 1L CT. In this setting, no prospective phase III data about the effect of chemotherapy has been provided to date. Only some retrospective data supports the use of 2L chemotherapy as it might provide disease control for selected patients (14–16). In our cohort, 21 patients (36.2%) received 2L CT, mainly with FOLFIRI (n=12, 57.1%). Reasons against 2L included early death after progressive disease under gemcitabine/platinum derivate (43.2%), reduced general condition (21.6%), patient’s wish (10.8%) or continuation of local therapy alone (13.5%).

Patients receiving 2L CT were in general about 11 years younger and achieved a mOS of 7.1 months vs. 2.9 months in the group without CT (p = 0.135). PFS under 2L CT was 3.3 months. These data are similar to the findings from the systematic review by Lamarca et al. on 2L CT in advance biliary cancer with mOS of 7.2 months and PFS of 3.2 months, calculated from first administration of 2L CT (29). Likewise, in the multicenter study with 174 patients published by Fornaro et al., patients achieved a mOS of 6.6 months and a PFS of 3.0 months with 2L CT (16). However, in this study, a wide variety of therapy regimens were used. The most frequent regimen used was a monotherapy with 5-FU or capecitabine in 28% of patients, while double or triple combinations were also applied. In another study by Lowery et al. with 124 patients also receiving 5-FU-based CT, a mOS of 11.0 months (95% CI: 8.8 – 13.1) was reported (30). The retrospective trial by Schweitzer et al. reported on 142 patients treated with 2L CT achieving a mOS of 9.9 months. In this cohort, 5-FU-based CT was also the most frequently used CT (70.4%) (15). Taking into account all the above mentioned data, our study reinforces the benefit of 2L 5-FU-based CT in patients with advanced cholangiocarcinoma.

At least eight patients (38.1%) reached 3L CT. These data are comparable to other studies (15). Reasons against 3L CT were, in most cases, a reduction of the general condition (69.23%), patient’s wish (15.38%), early death due to progressive disease (7.68%) and other reasons (7.69%).

Nevertheless, OS and PFS in biliary tract cancer are still disappointing.

More therapy options and further studies are needed. One therapy option is the triple combination with gemcitabine/cisplatin and nab-paclitaxel. A phase II study with 60 patients showed OS of 19.2 months and PFS of 11.8 months (31). A prospective phase III study, SWOG 1815, with exactly this combination is currently recruiting. However, the patients for the triple combination must show a very good performance status, which is not frequently found in patients in an advanced disease stage. A new and promising therapeutic approach is personalized medicine. Especially for iCCA, some targeted agents have been identified, which seem to prolong OS. The Fight 202-study showed a better clinical outcome in patients with iCCA and FGFR-2-fusions or -rearrangements when receiving pemigatinib (7). Furthermore, the use of ivosidenib in patients with IDH1-mutations, investigated in the ClarIDHy trial, a randomized, placebo controlled phase III study, showed increased median PFS (2.7 months vs. 1.4 months in the placebo group) and increased median OS (10.8 months vs. 9.7 months) (8). However, IDH1-mutations are detected in only 13-15% of patients with cholangiocarcinoma and FGFR-2 fusion in only about 13-15% of patients with iCCA. Thus, for the majority of patients, CT with gemcitabine/cisplatin and 5-FU-based 2L CT are still playing an important role in the therapy of advanced cholangiocarcinoma and may be an acceptable option for unselected patients, as confirmed in the present study.

As this is a single center study, a selection bias cannot be excluded. Other important limitations of our study are its retrospective design and the low statistical power due to the small number of patients included. Nevertheless, this retrospective design shows data in a real world setting in the treatment of palliative cholangiocarcinoma, where the balance between adherence to chemotherapy protocol in order to prolong survival and preserving of the health-related quality of life in patients with a poor overall prognosis remains a challenge. The strength of the present study is the analysis of the impact of dose reduction on OS, addressing the important question of the treating physician as to whether chemotherapy modification affects the outcome of cholangiocarcinoma patients.

In conclusion, our study shows that the combination of gemcitabine/platinum derivate is a feasible and tolerable therapy in unselected patients of daily clinical practice achieving similar OS and PFS as the randomized prospective phase II/III trials. However, the majority of patients needed therapy regimen adaptions, achieving also a clear benefit in terms of survival and a more acceptable tolerability. Thus, dose modifications or de-escalation should be evaluated during 1L CT with gemcitabine/platinum derivate to maintain quality of life for patients with very poor overall prognosis. Furthermore, our study reveals a potential survival benefit with 2L CT with FOLFIRI in selected patients, which should be evaluated in prospective trials.

## Data Availability Statement

The raw data supporting the conclusions of this article will be made available by the authors, without undue reservation.

## Ethics Statement

This retrospective study was reviewed and approved by the Ethics Committee of the Medical Faculty of the University of Bonn (No. 341/17). Written informed consent for participation was not required for this study in accordance with the national legislation and the institutional requirements.

## Author Contributions

CM: acquisition of data, analysis and interpretation of data, drafting of the manuscript, study concept and design. JF: acquisition of data, analysis and interpretation of data, drafting of the manuscript, study concept and design. RUM: acquisition of data, analysis and interpretation of data, drafting of the manuscript, study concept and design. FS: critical revision of the manuscript for important intellectual content. AB: critical revision of the manuscript for important intellectual content. RM: critical revision of the manuscript for important intellectual content. TZ: critical revision of the manuscript for important intellectual content. MM: critical revision of the manuscript for important intellectual content. GF: critical revision of the manuscript for important intellectual content. PB: critical revision of the manuscript for important intellectual content. MW: critical revision of the manuscript for important intellectual content. HM: critical revision of the manuscript for important intellectual content. SM: critical revision of the manuscript for important intellectual content. TG: critical revision of the manuscript for important intellectual content. JK: critical revision of the manuscript for important intellectual content. TW: critical revision of the manuscript for important intellectual content. CS: critical revision of the manuscript for important intellectual content. MG-C: acquisition of data, analysis and interpretation of data, drafting of the manuscript, study concept and design. All authors contributed to the article and approved the submitted version.

## Funding

This work was supported by the following grants awarded to MG-C: GO 1874/1-2 grant from the “Deutsche Forschungsgemeinschaft” (DFG), BONFOR from the University of Bonn, grant number 109255 from the “Deutsche Krebshilfe” (German Cancer Aid) and grant from the Reuthersche endowment fund of the University of Bonn.

## Conflict of Interest

Author MG-C has contributed to advisory boards for Roche, Eisai, MSD and AZ. However, these activities have no potential conflicts of interest with the manuscript.

The remaining authors declare that the research was conducted in the absence of any commercial or financial relationships that could be construed as a potential conflict of interest.

## Publisher’s Note

All claims expressed in this article are solely those of the authors and do not necessarily represent those of their affiliated organizations, or those of the publisher, the editors and the reviewers. Any product that may be evaluated in this article, or claim that may be made by its manufacturer, is not guaranteed or endorsed by the publisher.

## Abbreviations

5FU, 5-fluorouracil; CI, confidence interval; CTCAE, common terminology criteria for adverse events; ECOG, Eastern cooperative oncology group performance status; ERCP, endoscopic retrograde cholangiopancreatography; G, gemcitabine; GC, gemcitabine-cisplatin; HR, hazard ratio; IHD, ischemic heart disease; INR, international normalized ratio; mOS, median overall survival; n, number of patients; n.s., not significant; OS, overall survival; p, p-value; PDT, photodynamic therapy; PTC, percutaneous transhepatic cholangiography; RFA, radiofrequency ablation; SC, systemic chemotherapy; SIRT, selective internal radiation therapy; TACE, transcatheter arterial chemoembolization; γGT, gamma-glutamyl transferase.
